# Sexual activity in a large representative cohort of Polish men: Frequency, number of partners, correlates, and quality of life

**DOI:** 10.1371/journal.pone.0296449

**Published:** 2024-01-19

**Authors:** Mikolaj Przydacz, Marcin Chlosta, Adrian Andrzej Chrobak, Pawel Rajwa, Przemyslaw Dudek, Tomasz Wiatr, Katarzyna Gronostaj, Anna Katarzyna Czech, Marcin Miszczyk, Michal Skalski, Dominika Dudek, Piotr Chlosta

**Affiliations:** 1 Department of Urology, Jagiellonian University Medical College, Krakow, Poland; 2 Department of Adult Psychiatry, Jagiellonian University Medical College, Krakow, Poland; 3 Department of Urology, Medical University of Silesia, Zabrze, Poland; 4 Department of Urology, Comprehensive Cancer Center, Medical University of Vienna, Vienna, Austria; 5 Collegium Medicum Faculty of Medicine, WSB University, Dabrowa Gornicza, Poland; Indiana University School of Medicine, UNITED STATES

## Abstract

**Introduction:**

Sexual activity of men has been evaluated at the population-level in different regions of the world. However, reliable data are lacking for Eastern Europe. Therefore, the aim of this study was to analyze the frequency of sexual activity and the number of sexual partners in a large representative cohort of Polish men.

**Methods:**

We performed a cross-sectional investigation with computer-assisted web interviews. Participants were stratified by age (≥18 years) and place of residence. The most recent population census was used to produce a population-representative sample of respondents. Men’s sexual activity was then correlated with multiple variables.

**Results:**

We enrolled 3001 men, representative for age and place of residence, including adequate proportions of respondents from urban and rural areas. Most Polish men were sexually active, predominantly having had sex at least weekly with one partner. Almost 18% of respondents declined sexual intercourse and/or sexual partner in the prior year. The highest sexual activity was observed for men 35-44-years-old (for sex frequency) and 18-24-years-old (for partner number), living in medium-sized cities, employed, and married (for sex frequency) or divorced (for partner number). Erectile dysfunction negatively affected the frequency of sexual activity and lowered the number of sexual partners, although premature ejaculation did not have any effect. Frequency of sexual activity and number of sexual partners correlated well with psychological distress, quality of sex life, and overall life quality. Whereas lifestyle habits including smoking and alcohol intake decreased the likelihood of sexual activity, all analyzed comorbidities did not affect sex life.

**Conclusions:**

This study of men’s sexual activity was the first population-representative and nationwide investigation performed in Poland. Most Polish men were sexually active and sexual activity correlated with multiple variables including sociodemographic factors, erectile functioning, mental distress, overall and sex-specific quality of life, and lifestyle habits.

## Introduction

Healthy sexual functioning of men is a significant part of life with an important effect on overall well-being. Frequent sexual activity is associated with many benefits for physical and mental health, including a reduction in cardiovascular incidents, reduced risk of fatal coronary events or prostate cancer, and better life satisfaction or quality of life [1]. Some studies even suggest a bidirectional relationship between frequent sexual activity and good mental and physical health. Conversely, infrequent sexual activity may contribute to poor health, diminished self-esteem, and increased mortality [2].

The frequency of men’s sexual activity has been evaluated in several large epidemiological studies. These studies yielded disparate results with up to 95% of men characterized as sexually active across different age groups [3–5]. Importantly, regional differences in sexual behavior have been noted, and experts stipulate that inter- and intracultural factors are largely responsible for these differences [6]. Possible cultural variables include traditions, religion-based norms, changes in gender roles, differences in gender equality, and restrictions on sexuality [6].

However, reliable data for men’s sexual activity are lacking for Central and Eastern Europe. Even in large-scale and population-level European studies conducted to ascertain this issue, investigators have not included countries from Central and Eastern Europe [5–8]. To date, no large population-representative study in any country of this region has been performed to reliably evaluate men’s sexual activity. Poland, the largest country in Central Europe [9, 10], and by land area the third largest in Eastern Europe after Russia and Ukraine [11], is no exception. Importantly, Poland and other Central and Eastern European countries have unique demographics, e.g., homogeneity or supra-ethnic uniformity; in Poland, ≥ 99% of residents are Caucasian and ≥ 90% of residents are of Polish identity [12]. Such unique demographic features need to be considered when discussing population-based studies for any set of symptoms or disorders. Because Central-Eastern Europe is often considered a distinct cultural entity and Slavic people are culturally different from other European people [13], sexual behavior may differ significantly when compared with other ethnic groups. Indeed, some local cultural norms may have significant effects on social- and health-related activities, especially for sensitive area of sexual life. In addition, with a relatively high number of people living in Polish rural regions, available foreign data on men’s sexual activity may not be fully transferable to Poland because most epidemiological data for sexual behaviors do not include comparisons between urban and rural areas. Considering all these factors, we have limited understanding of the sexual behavior of men in Poland and lack reliable population-level estimates. These estimates attract interdisciplinary frameworks for national health improvement programs and allocation of appropriate resources by healthcare systems. Importantly, epidemiological data and large datasets have clear benefits to public health. Therefore, the aim of this study was to evaluate the frequency of sexual activity and number of sexual partners in a large representative cohort of Polish men aged ≥18 years in all geographical regions of Poland. We then examined factors associated with sexual frequency and partner number that included sociodemographic parameters, erectile and ejaculatory functioning, psychological distress, comorbidities, lifestyle habits, overall quality of life, and treatment-related behavior.

## Methods

We extracted data from the ED POLAND study, a population-based, representative, and cross-sectional investigation designed to ascertain sexual activity and sexual dysfunction of Polish men. Standardized guidelines and well-established recommendations for reporting observational studies were followed [14]. The study was approved by the research ethics committee of Jagiellonian University Medical College, Krakow, Poland (1072.6120.331.2021) and registered with ClinicalTrials.gov (NCT05462171). All participants provided informed consent to be included in the study.

### Design

This study was performed with computer-assisted web interviews (CAWI) stratified by age and place of residence (i.e., quota controls). The study included respondents from all geographical regions of Poland (i.e., from all 16 states/voivodships), with adequate numbers of participants from urban and rural areas. Urban and rural areas were defined according to the definitions of the Central Statistical Office of Poland [15]. The most recent population census (2021) was used to produce a population-representative sample of respondents [16]. This sampling ensured that the data collected were representative of the general population.

### Survey distribution

The survey was distributed by IPSOS Poland, a research agency with relevant quality certificates (OFBOR, ESOMAR, PKJPA, PKJBI) between November and December 2022. The survey participants were extracted by quota controls from a pre-existing Internet IPSOS panel. An e-mail was sent to 5800 members to invite them to participate in a confidential survey. Each respondent received a unique Uniform Resource Locator (URL), and all responses were collected on a web server with appropriate Secure Sockets Layer (SSL) certificate. Note: In 2022, 93.33% of households in Poland had Internet access, with no significant difference between urban and rural areas [17]. There were regular quality-control and stratification checks.

### Measures

For frequency of sexual activity, we asked the following question: About how often did you have sex during the last 12 months?’ Answers were ‘not at all’, ‘less than once a month’, ‘once a month’, ‘2–3 times a month’, ‘weekly’, ‘2–3 times a week’, ‘≥4 times a week’, ‘hard to say/do not know’. For number of sexual partners, we used the following question: ‘How many sex partners have you had in the last 12 months?’; Answers were ‘no partners’, ‘1 partner’, ‘2 partners’, ‘3 partners’, ‘4 partners’, ‘5–10 partners’, ‘≥10 partners’, ‘hard to say/do not know’. These two questions were adapted from a nationally representative US survey, the General Social Survey, to make our results reliably comparable with this survey and other large-scale population-based analyses on male sexual activity [3]. Because of low numbers of participants endorsing some response options, we combined proximal categories, leaving four groups for each analysis: for frequency, not at all, less than once a month, 1–3 times per month, weekly or more; for partners, no partners, 1 partner, 2 partners, 3 or more partners.

For each respondent, we collected general demographic data, including age (age groups: 18–24, 25–34, 35–44, 45–54, 55–64, ≥65), place of residence (city with >500,000 inhabitants, city with 100,000–500,000 inhabitants, city with 20,000–100,000 inhabitants, city with <20,000 inhabitants, rural areas), level of education (elementary, vocational, secondary, higher), employment status (employed, unemployed, pensioner, other), and marital status (single, married or in a relationship, divorced or separated, widower).

In our study, we also used the five-item International Index of Erectile Function (IIEF-5) to assess erectile dysfunction (ED). An IIEF-5 score of 16 or less was a referent for ED, with further analyses of different levels of ED severity based on IIEF score: 22–25 without ED; 17–21 mild ED; 12–16 mild to moderate ED; 8–11 moderate ED; 5–7 severe ED [18]. The Premature Ejaculation Diagnostic Tool (PEDT) was used to evaluate premature ejaculation (PE); a PEDT score of 11 or more was a referent for PE, with further analyses of different scale’s cutoff points: ≤8 without PE; 9–10 probable presence of PE; ≥11 presence of PE [19]. The Hospital Anxiety and Depression Scale (HADS) was used to investigate psychological distress. A total HADS score of 0–16 indicated no distress, 17–22 borderline distress, and 23–48 significant distress [20]. All three questionnaires were rigorously translated, adapted, and validated for Polish versions [21–24].

Participants were further asked about relevant comorbidities (i.e., arterial hypertension, myocardial infarction, any cardiac disease, diabetes, overweight, lipid disorders, stroke, any pulmonary disease, any surgeries in abdomen or pelvis) and lifestyle habits (i.e., smoking, alcohol intake). All comorbidities were self-reported; no attempts were made to validate the respondents’ answers with medical records. Then, we inquired respondents about their overall (‘If you were spend the rest of your life in your current condition, how would you describe your overall well-being?’) and sex-specific (‘In the past 4 weeks, how were you satisfied with your sex life?’) quality of life. Finally, the respondents were asked about treatment-related behavior for their sex life (treatment seeking, receiving, satisfaction, and continuation).

### Statistics

We calculated a sample size with the methodology that was used in other population-based studies of men’s sexual health [25–28]. The sample size for our study depended on the underlying event rate in the population, population standard deviation, acceptable level of significance, expected effect size, and power of the study [29]. The sample size was calculated before initiating a study and was not changed during the study course. On the basis of the population age distribution and recommendations from the recent census for future population-representative analyses in Poland, we set the sample size to 3000 respondents. With a national sample of 3000, there was a 95% certainty that the overall survey results were between ± 1–2% of what they would have been had we polled the entire adult male Polish population.

For quantitative (ordinal) variables, we used nonparametric tests: Mann-Whitney or Kruskal-Wallis with post-hoc Dunn test, if applicable. For qualitative (categorical) variables, we used chi-square or exact Fisher test, if low expected counts.

We also used multivariable logistic regression models and presented results as odd ratios (ORs) with a 95% confidence interval (CI). We defined a dependent variable as no sexual activity. An alternative approach, with a dependant variable defined as number of sexual partners, i.e., no sexual partners, was dismissed because of highly similar results, i.e., no sexual activity almost always meant no sexual partners. For independent variables, all potential predictors were included in the regression (the predictors were not selected) because we observed a high ratio of the number of observations to the number of variables (i.e., events per variable) of approximately 10 (i.e., the generally accepted threshold) [30]. The results of standardized tools were entered in the models as raw data (raw scores).

A p-value less than 0.05 was considered to be statistically significant. R (R Core Team, version 4.3.0, 2023. R: A language and environment for statistical computing. R Foundation for Statistical Computing, Vienna, Austria) was used to conduct data analysis.

## Results

In this study, we included 3001 men, representative for age and place of residence. Most of the participants had at least secondary education (n = 2521; 84%), were employed (n = 2199; 73.3%), and married (n = 1964; 65.4%). More respondents lived in urban areas than in rural regions (n = 2274 vs. n = 727; 75.8% vs. 24.2%). The response rate of our survey was 51.7%.

**Fig 1:**
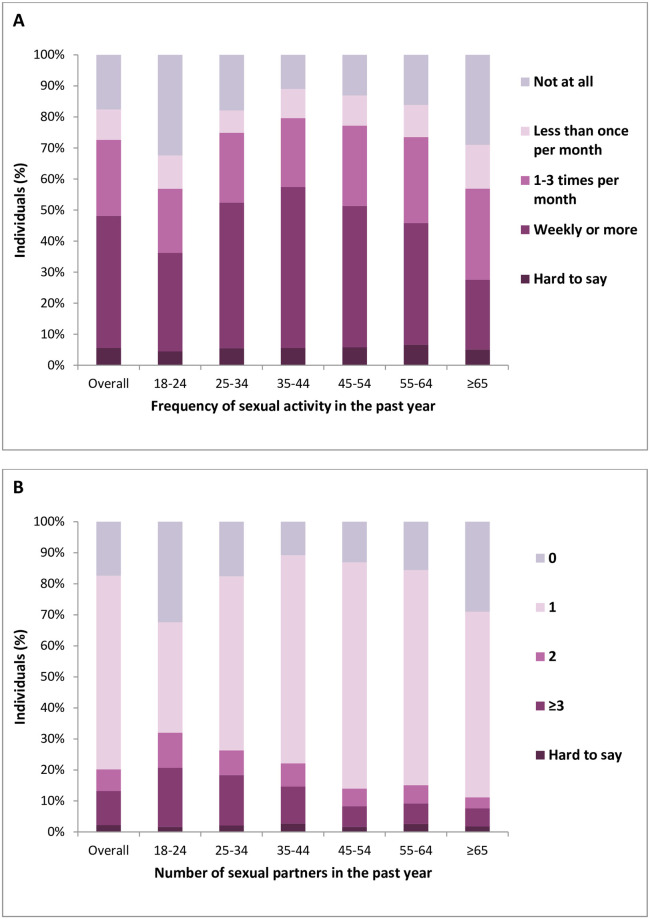
Frequency of sexual activity (A) and number of sexual partners (B) overall and in all age groups.

Overall, most men were sexually active during the preceding 12 months, with most (42.55%) having had sex weekly or more often (Fig 1, S1 Table). Almost 18% of the respondents did not have sex during the last year.

In general, most men (62.55%) had one sexual partner and 17.4% of respondents did not have a partner in the past year (Fig 1, S1 Table).

### Age

The highest frequency of sexual intercourse was observed with 35-44-year-old men and the lowest with 18–24 and ≥65 years-old (p<0.001, Fig 1, S1 Table). Therefore, the frequency of sexual activity among all age groups presented as an inverted “U” shape.

The highest number of sexual partners was observed with 18-24-year-old men and the lowest with ≥65 years-old (p<0.001, Fig 1, S1 Table). Thus, with increasing age, we observed a gradual increase in number of men who reported one sexual partner.

### Place of residence

The highest frequency of sexual activity was observed for residents of medium-sized cities with 20,000–100,000 inhabitants; the lowest activity was for men from rural areas and small-sized cities with less than 20,000 inhabitants (p = 0.013, Table 1). Similar differences were observed for number of sexual partners, i.e., the highest number of sexual partners was for men from medium-sized cities and the lowest number of sexual partners was for men from rural areas and small-sized cities (p = 0.004, Table 1).

**Table 1:** Frequency of sexual activity and number of sexual partners based on place of residence.

Parameter	Value	Place of residence					p
		City with >500,000 inhabitants (N = 424)—A	City with 100,000–500,000 inhabitants (N = 629)—B	City with 20,000–100,000 inhabitants (N = 742)—C	City with <20,000 inhabitants (N = 479)—D	Rural areas (N = 727)—E	
Frequency of sexual activity in the past year	Not at all	72 (16.98%)	112 (17.81%)	107 (14.42%)	97 (20.25%)	139 (19.12%)	p = 0.013
	Less than once per month	49 (11.56%)	51 (8.11%)	71 (9.57%)	50 (10.44%)	72 (9.90%)	B>D C>E,D
	1–3 times per month	93 (21.93%)	151 (24.01%)	188 (25.34%)	125 (26.10%)	178 (24.48%)	
	Weekly or more	187 (44.10%)	284 (45.15%)	336 (45.28%)	182 (38.00%)	288 (39.61%)	
	Hard to say	23 (5.42%)	31 (4.93%)	40 (5.39%)	25 (5.22%)	50 (6.88%)	
Number of sexual partners in the past year	0	70 (16.51%)	95 (15.10%)	104 (14.02%)	106 (22.13%)	146 (20.08%)	p = 0.004
	1	278 (65.57%)	419 (66.61%)	465 (62.67%)	278 (58.04%)	437 (60.11%)	C>B,A,D,E
	2	22 (5.19%)	40 (6.36%)	64 (8.63%)	35 (7.31%)	48 (6.60%)	
	≥3	42 (9.91%)	59 (9.38%)	97 (13.07%)	55 (11.48%)	76 (10.45%)	
	Hard to say	12 (2.83%)	16 (2.54%)	12 (1.62%)	5 (1.04%)	20 (2.75%)	

p—Kruskal-Wallis test + post-hoc test (Dunn test)

We did not find any differences in frequency of sexual intercourse and number of sexual partners across all 16 states/voivodships of Poland (S2 Table).

### Education

**Fig 2:**
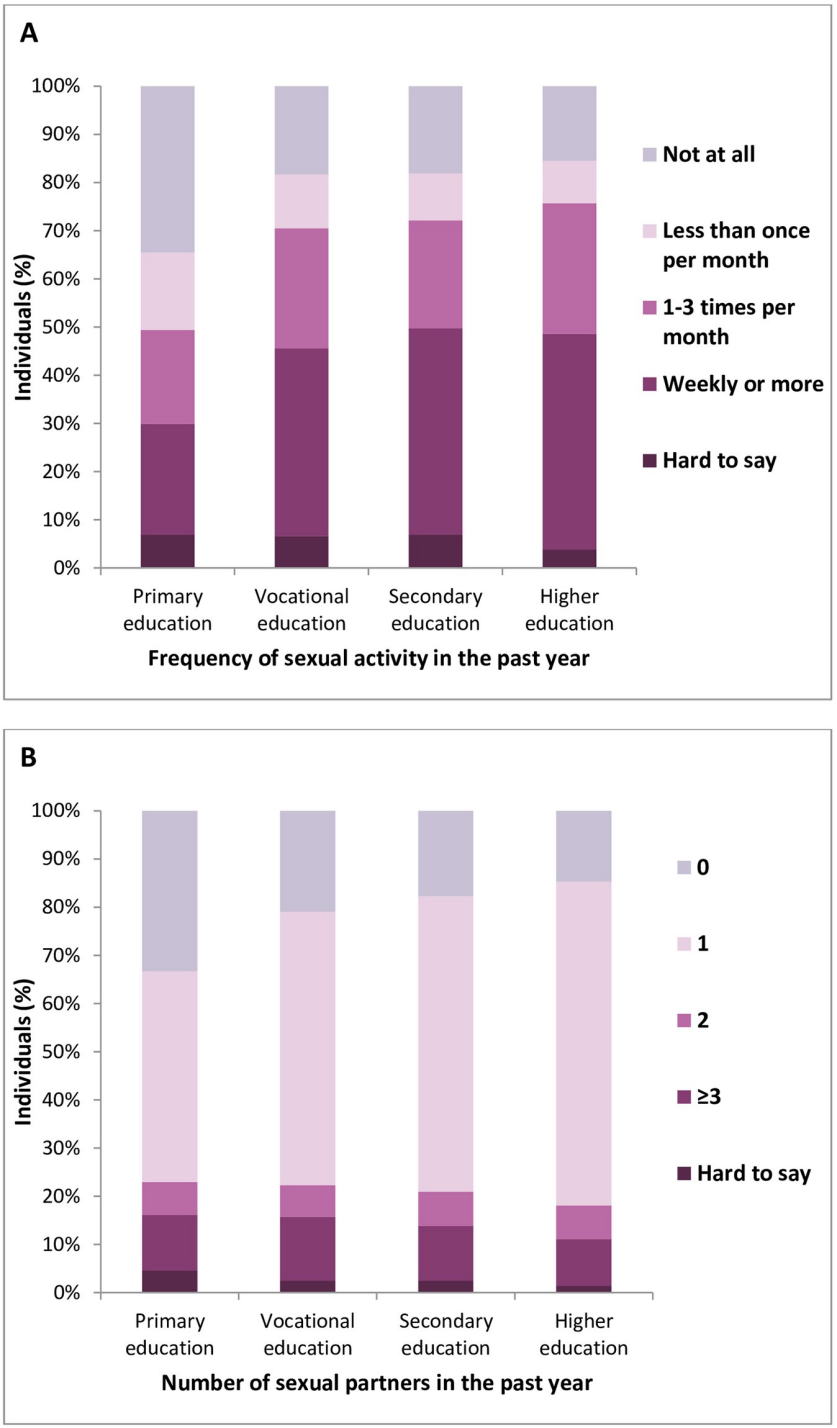
Frequency of sexual activity (A) and number of sexual partners (B) as a function of education level.

Men with higher education were the most sexually active, whereas men with only elementary education had the lowest sexual activity (p<0.001, Fig 2, S3 Table). There were no statistical differences in number of sexual partners among the respondents in terms of education level (p = 0.083, Fig 2, S3 Table).

### Employment status

Employed men had the highest sexual activity and unemployed respondents were the least sexually active (p<0.001, S4 Table). Similarly, employed men had more sexual partners than the unemployed, but pensioners had the lowest number of sexual partners (p<0.001, S4 Table).

### Marital status

The highest sexual activity was observed in men who were married or in a relationship, and the lowest sexual activity was characteristic for singles (p<0.001, Table 2). Conversely, the highest number of sexual partners in the last 12 months was associated with men who were divorced or separated, and widowers had the lowest number of partners (p<0.001, Table 2).

**Table 2:** Frequency of sexual activity and number of sexual partners based on marital status.

Parameter	Value	Marital status				p
		Single (N = 833)—A	Married or in a relationship (N = 1964)—B	Divorced or separated (N = 156)—C	Widower (N = 48)—D	
Frequency of sexual activity in the past year	Not at all	337 (40.46%)	143 (7.28%)	32 (20.51%)	15 (31.25%)	p<0.001
	Less than once per month	82 (9.84%)	190 (9.67%)	17 (10.90%)	4 (8.33%)	C>A B>C,D,A
	1–3 times per month	147 (17.65%)	535 (27.24%)	46 (29.49%)	7 (14.58%)	
	Weekly or more	209 (25.09%)	1003 (51.07%)	49 (31.41%)	16 (33.33%)	
	Hard to say	58 (6.96%)	93 (4.74%)	12 (7.69%)	6 (12.50%)	
Number of sexual partners in the past year	0	331 (39.74%)	137 (6.98%)	37 (23.72%)	16 (33.33%)	p<0.001
	1	223 (26.77%)	1569 (79.89%)	62 (39.74%)	23 (47.92%)	C,B>A,D
	2	87 (10.44%)	101 (5.14%)	18 (11.54%)	3 (6.25%)	
	≥3	157 (18.85%)	137 (6.98%)	32 (20.51%)	3 (6.25%)	
	Hard to say	35 (4.20%)	20 (1.02%)	7 (4.49%)	3 (6.25%)	

p—Kruskal-Wallis test + post-hoc test (Dunn test)

### Erectile functioning

**Fig 3:**
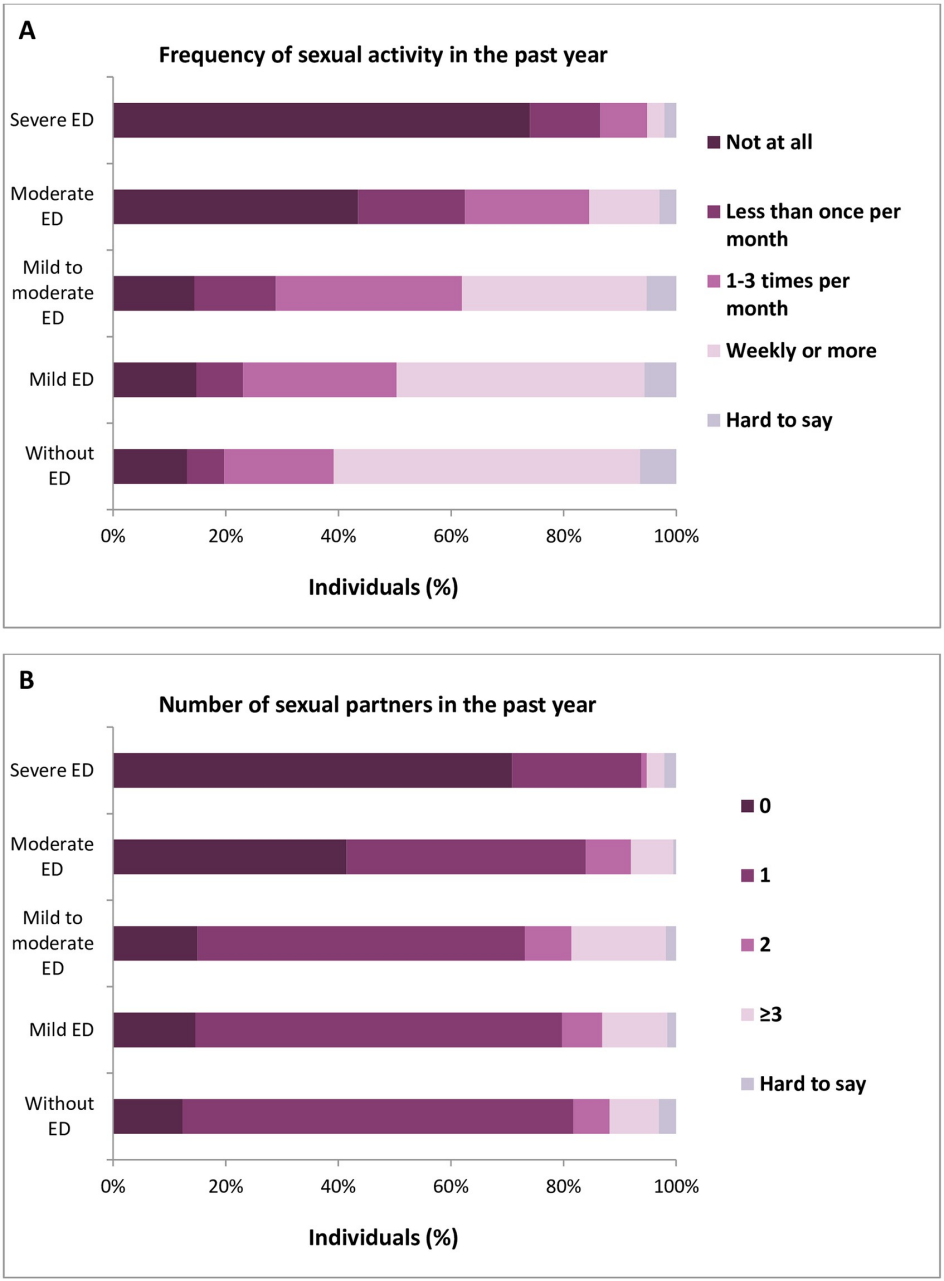
Frequency of sexual activity (A) and number of sexual partners (B) as a function of different levels of ED severity based on IIEF-5 score (22–25 without ED; 17–21 mild ED; 12–16 mild to moderate ED; 8–11 moderate ED; 5–7 severe ED).

Erectile functioning had a profound effect on both frequency of sexual activity and number of sexual partners. With the IIEF-5 score of 16 or less for determining ED, we found that frequency of sexual activity of men with no ED was higher than for men with ED (p<0.001, Table 3). Similarly, the number of sexual partners in the last 12 months was significantly higher in the group of respondents with mild to no ED (IIEF score ≥17; p<0.001, Table 3). In further analysis, with different levels of ED severity based on IIEF-5, we also investigated correlations between both the frequency of sexual activity/number of sexual partners and the ED severity (i.e., the more severe ED, the lower sexual activity/the lower number of sexual partners, p<0.001, Fig 3, S5 Table).

**Table 3:** Frequency of sexual activity and number of sexual partners based on erectile functioning (IIEF-5 score of 16 or less was a reference for erectile dysfunction).

Parameter	Value	IIEF score		p
		IIEF: 16 points and less (N = 902)	IIEF: 17 points and more (N = 2099)	
Frequency of sexual activity in the past year	Not at all	245 (27.16%)	282 (13.43%)	p<0.001
	Less than once per month	138 (15.30%)	155 (7.38%)	
	1–3 times per month	252 (27.94%)	483 (23.01%)	
	Weekly or more	227 (25.17%)	1050 (50.02%)	
	Hard to say	40 (4.43%)	129 (6.15%)	
Number of sexual partners in the past year	0	242 (26.83%)	279 (13.29%)	p<0.001
	1	459 (50.89%)	1418 (67.56%)	
	2	67 (7.43%)	142 (6.77%)	
	≥3	120 (13.30%)	209 (9.96%)	
	Hard to say	14 (1.55%)	51 (2.43%)	

p—Mann-Whitney test

### Ejaculatory functioning

In our study, ejaculatory functioning in terms of premature ejaculation had no effect on frequency of sexual activity and number of sexual partners (with a referent PEDT score of 11 or more for PE: p = 0.184 and p = 0.761, for sex frequency and number of sex partners, respectively, S6 Table; with different scale’s cutoff points of PEDT: p = 0.069 and p = 0.061, for sex frequency and number of sex partners, respectively, S7 Table).

### Psychological distress

**Fig 4:**
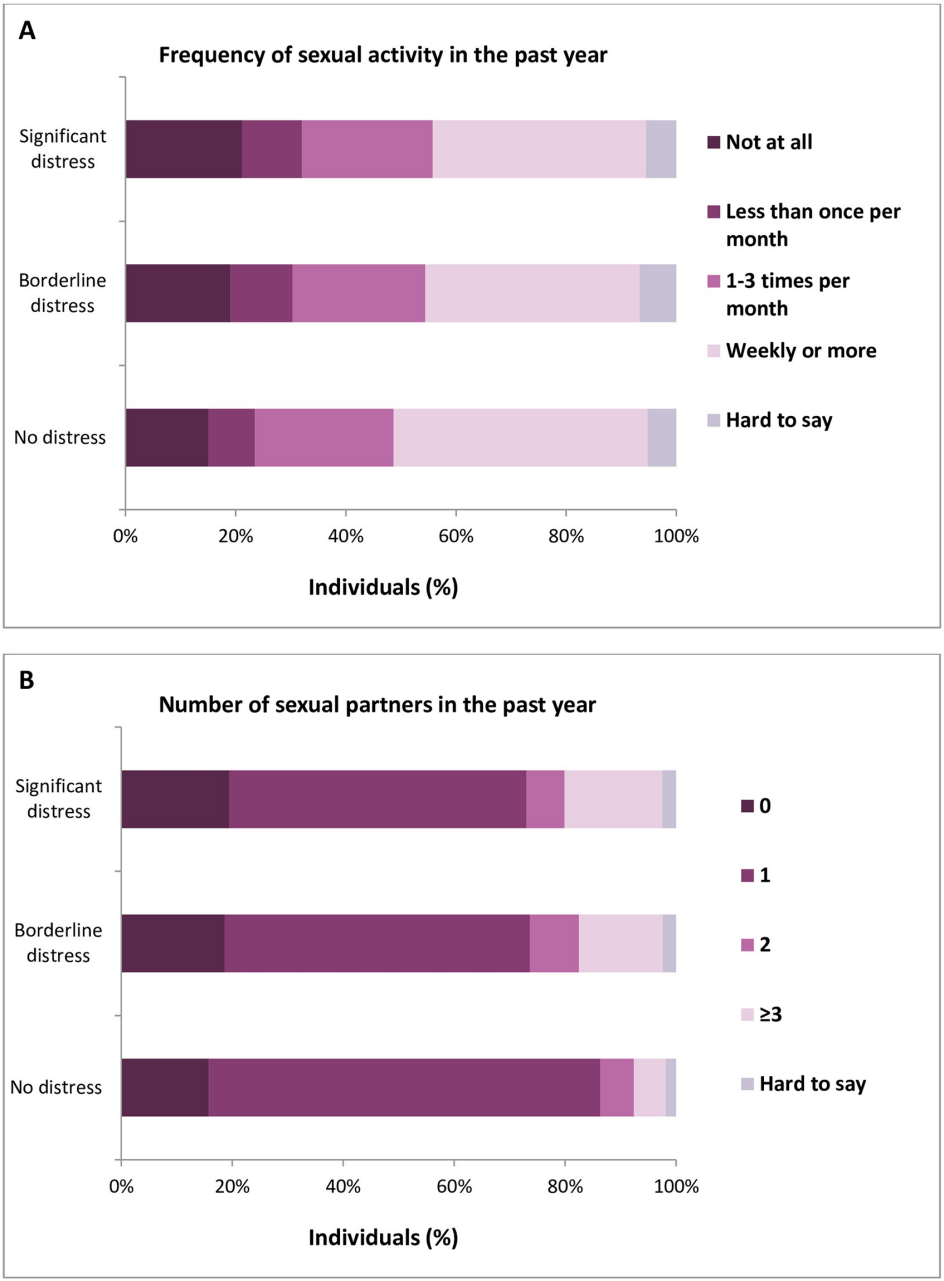
Frequency of sexual activity (A) and number of sexual partners (B) as a function of psychological distress based on HADS score (0–16 no distress, 17–22 borderline distress, and 23–48 significant distress).

We discovered that the frequency of intercourse was significantly higher in the group of men without psychological distress than for participants with borderline or significant stress (total HADS score, p<0.001, Fig 4, S8 Table). Conversely, the number of sexual partners in the last year was significantly higher for men with borderline or significant distress compared with respondents without the condition (total HADS score, p<0.001, Fig 4, S8 Table).

### Effect of frequency and partner number on sex-specific and overall quality of life

Responses to the question ‘In the past 4 weeks, how were you satisfied with your sex life?’ demonstrated that frequency of sexual activity and number of sexual partners had significant effects on the quality of sex life. Our analysis showed that the higher the frequency of sexual activity and the higher the number of sexual partners, the higher the quality of sex life (p<0.001; Table 4).

**Table 4:** Correlations between frequency of sexual activity/number of sexual partners and overall/sex-specific quality of life.

**Parameter**	**Value**	**Sex-specific quality of life**							**p**
		**Satisfied** **(N = 806)—A**		**Moderately satisfied** **(N = 959)—B**	**Mixed** **(N = 653)—C**	**Moderately dissatisfied** **(N = 268)—D**		**Dissatisfied** **(N = 315)—E**	
Frequency of sexual activity in the past year	Not at all	66 (8.19%)		90 (9.38%)	117 (17.92%)	78 (29.10%)		176 (55.87%)	p<0.001
	Less than once per month	34 (4.22%)		74 (7.72%)	79 (12.10%)	44 (16.42%)		62 (19.68%)	A>B>C>D>E
	1–3 times per month	134 (16.63%)		295 (30.76%)	199 (30.47%)	65 (24.25%)		42 (13.33%)	
	Weekly or more	519 (64.39%)		458 (47.76%)	220 (33.69%)	59 (22.01%)		21 (6.67%)	
	Hard to say	53 (6.58%)		42 (4.38%)	38 (5.82%)	22 (8.21%)		14 (4.44%)	
Number of sexual partners in the past year	0	80 (9.93%)		91 (9.49%)	117 (17.92%)	67 (25.00%)		166 (52.70%)	p<0.001
	1	537 (66.63%)		677 (70.59%)	382 (58.50%)	155 (57.84%)		126 (40.00%)	A>C,D,E B,C>D,E D>E
	2	55 (6.82%)		77 (8.03%)	58 (8.88%)	10 (3.73%)		9 (2.86%)	
	≥3	104 (12.90%)		102 (10.64%)	82 (12.56%)	34 (12.69%)		7 (2.22%)	
	Hard to say	30 (3.72%)		12 (1.25%)	14 (2.14%)	2 (0.75%)		7 (2.22%)	
		**Overall quality of life**							**p**
		**Delighted** **(N = 392)—A**	**Pleased** **(N = 766)—B**	**Mostly satisfied** **(N = 645)—C**	**Mixed** **(N = 613)—D**	**Mostly dissatisfied** **(N = 339)—E**	**Unhappy** **(N = 182)—F**	**Terrible** **(N = 64)—G**	
Frequency of sexual activity in the past year	Not at all	56 (14.29%)	73 (9.53%)	90 (13.95%)	119 (19.41%)	92 (27.14%)	61 (33.52%)	36 (56.25%)	p<0.001
	Less than once per month	21 (5.36%)	41 (5.35%)	74 (11.47%)	67 (10.93%)	51 (15.04%)	31 (17.03%)	8 (12.50%)	B,A>C,D>E>F>G
	1–3 times per month	60 (15.31%)	182 (23.76%)	196 (30.39%)	165 (26.92%)	80 (23.60%)	40 (21.98%)	12 (18.75%)	
	Weekly or more	223 (56.89%)	431 (56.27%)	252 (39.07%)	226 (36.87%)	100 (29.50%)	39 (21.43%)	6 (9.38%)	
	Hard to say	32 (8.16%)	39 (5.09%)	33 (5.12%)	36 (5.87%)	16 (4.72%)	11 (6.04%)	2 (3.12%)	
Number of sexual partners in the past year	0	61 (15.56%)	75 (9.79%)	94 (14.57%)	118 (19.25%)	80 (23.60%)	56 (30.77%)	37 (57.81%)	p<0.001
	1	241 (61.48%)	548 (71.54%)	421 (65.27%)	366 (59.71%)	188 (55.46%)	97 (53.30%)	16 (25.00%)	B>D,E,F,G C>E,F,G A,D,E>F,G F>G
	2	24 (6.12%)	49 (6.40%)	54 (8.37%)	45 (7.34%)	21 (6.19%)	14 (7.69%)	2 (3.12%)	
	≥3	49 (12.50%)	77 (10.05%)	68 (10.54%)	73 (11.91%)	43 (12.68%)	12 (6.59%)	7 (10.94%)	
	Hard to say	17 (4.34%)	17 (2.22%)	8 (1.24%)	11 (1.79%)	7 (2.06%)	3 (1.65%)	2 (3.12%)	

p—Kruskal-Wallis test + post-hoc analysis (Dunn test)

With the question ‘If you were spend the rest of your life in your current condition, how would you describe your overall well-being?’, we also observed positive correlations between the frequency of sexual activity/number of sexual partners and overall quality of life. We demonstrated that the higher the frequency of sexual activity and the higher number of sexual partners, the higher the overall quality of life (p<0.001; Table 4).

### Treatment-related behavior

When we asked ‘Have you ever sought medical treatment for problems with your sex life’, 11.1% (n = 334) of respondents were looking for such help. We found that men with three or more partners in the preceding year were the most active in seeking treatment, whereas men who had one partner were the least likely to seek treatment (p<0.001, Table 5). Further, men who had intercourse less than once per month were the most likely to be looking for treatment, and men who had sex at least once a week were the least likely to seek treatment (p<0.001, Table 5).

**Table 5:** Treatment seeking for problems with sex life.

Parameter	Group	Treatment seeking for problems with sex life		p
		Yes	No	
Frequency of sexual activity in the past year	Not at all (N = 527)	57 (10.82%)	470 (89.18%)	p<0.001
	Less than once per month (N = 293)	47 (16.04%)	246 (83.96%)	
	1–3 times per month (N = 735)	104 (14.15%)	631 (85.85%)	
	Weekly or more (N = 1277)	108 (8.46%)	1169 (91.54%)	
Number of sexual partners in the past year	0 (N = 521)	53 (10.17%)	468 (89.83%)	p<0.001
	1 (N = 1877)	161 (8.58%)	1716 (91.42%)	
	2 (N = 209)	36 (17.22%)	173 (82.78%)	
	≥3 (N = 329)	74 (22.49%)	255 (77.51%)	

p—chi-square or exact Fisher test

We did not find significant correlations between treatment receiving, treatment satisfaction, and treatment continuation with frequency of sexual activity/number of sexual partners.

### Multivariate analysis

Table 6 presents the results of our multivariable logistic regression analysis for sexual activity. The regression model confirmed the effects of age, place of residence, education, employment, and marital status as well as correlations with erectile dysfunction, mental distress, sex-specific and overall quality of life. Whereas no comorbidity influenced the frequency of sexual activity, lifestyle habits including smoking (OR 1.313; CI 1.004–1.718; p = 0.047) and alcohol intake (OR 1.572; CI 1.079–2.291; p = 0.019) decreased the likelihood of sexual activity.

**Table 6:** The multivariable logistic regression analysis for frequency of sexual activity.

Variable		OR	95%CI		p
Age	18–24	1	ref.		
	25–34	0,964	0,632	1,472	0,866
	35–44	0,91	0,579	1,43	0,682
	45–54	1,765	1,073	2,903	0,025 *
	55–64	1,982	1,208	3,254	0,007 *
	≥65	2,743	1,458	5,159	0,002 *
Place of residence	City with >500,000 inhabitants	1	ref.		
	City with 100,000–500,000 inhabitants	1,235	0,81	1,882	0,327
	City with 20,000–100,000 inhabitants	0,545	0,354	0,89	0,021 *
	City with <20,000 inhabitants	1,005	0,644	1,569	0,982
	Rural areas	1,108	0,731	1,681	0,629
Education	Elementary	1	ref.		
	Vocational	0,491	0,251	0,96	0,037 *
	Secondary	0,679	0,366	1,259	0,219
	Higher	0,708	0,373	1,346	0,293
Employment status	Employed	1	ref.		
	Unemployed	2,292	1,6	3,284	<0,001 *
	Pensioner	1,744	1,173	2,593	0,006 *
	Other	0,912	0,42	1,982	0,817
Marital status	Single	1	ref.		
	Married or living with a partner	0,067	0,048	0,094	<0,001 *
	Divorced or separated	0,181	0,106	0,31	<0,001 *
	Widower	0,336	0,153	0,739	0,007 *
IIEF	[score]	0,939	0,916	0,963	<0,001 *
PEDT	[score]	1,034	0,922	1,077	0,114
HADS	[score]	0,951	0,922	0,981	0,001 *
Sex-specific quality of life	Satisfied	1	ref.		
	Moderately satisfied	1,599	1,024	2,496	0,039 *
	Mixed	3,47	2,157	5,584	<0,001 *
	Moderately dissatisfied	7,903	4,589	13,612	<0,001 *
	Dissatisfied	25,25	14,684	43,418	<0,001 *
Overall quality of life	Delighted	1	ref.		
	Pleased	0,669	0,406	1,101	0,113
	Mostly satisfied	0,568	0,335	0,963	0,036 *
	Mixed	0,729	0,422	1,26	0,258
	Mostly dissatisfied	0,562	0,311	0,815	0,001*
	Unhappy	0,518	0,264	0,799	0,001*
	Terrible	0,591	0,255	1,374	0,222
Diabetes	Yes	1	ref.		
	No	1,221	0,793	1,881	0,365
Any pulmonary disease	Yes	1	ref.		
	No	1,276	0,775	2,099	0,338
Any cardiac disease	Yes	1	ref.		
	No	0,83	0,499	1,382	0,474
Arterial hypertension	Yes	1	ref.		
	No	1,303	0,912	1,861	0,146
Lipid disorders	Yes	1	ref.		
	No	0,891	0,626	1,269	0,523
Myocardial infarction	Yes	1	ref.		
	No	0,944	0,531	1,677	0,844
Stroke	Yes	1	ref.		
	No	0,698	0,376	1,295	0,254
Smoking	Yes	1	ref.		
	No	1,313	1,004	1,718	0,047 *
Obesity	Yes	1	ref.		
	No	0,984	0,744	1,3	0,908
Alcohol intake (≥2 drinks per day)	Yes	1	ref.		
	No	1,572	1,079	2,291	0,019 *
Any surgeries in abdomen or pelvis	Yes	1	ref.		
	No	1,087	0,737	1,602	0,674

p—multivariate logistic regression

* staistical significance (p<0.05)

## Discussion

Sexual health has been increasingly investigated from public health perspectives. Our study is the first in Central and Eastern Europe that reliably analysed men’s sexual activity, i.e., frequency of sexual activity and number of sexual partners, at the population level. The analysis included a representative group of men aged at least 18 years from all geographical regions of Poland, with adequate proportions of urban and rural area participants. We used widely accepted survey instruments for assessment of erectile dysfunction, premature ejaculation, and psychological distress. Finally, we analysed many covariates, including comorbidities, lifestyle habits, effect on quality of life, and treatment-related behaviors.

We found that Polish men were sexually active, with most men having had sex at least once per week with one partner in the past 12 months. The highest frequency of sexual activity was observed for 35-44-year-old men, living in medium-sized cities with 20,000–100,000 inhabitants, who had higher education, were employed, and married or in a relationship. The lowest frequency of sexual intercourse was noted for men aged ≥65 years, from rural areas and small-sized cities with less than 20,000 inhabitants, with elementary education, unemployed, or single. Further, men with the highest number of sexual partners were 18-24-years-old, living in medium-sized cities with 20,000–100,000 inhabitants, employed, divorced, or separated. The lowest number of sexual partners was observed for men aged ≥65 years, from rural areas and small-sized cities with less than 20,000 inhabitants, unemployed, or widowed.

Ueda et al. recently described trends in frequency of sexual activity and number of sexual partners among American men aged 18 to 44 years [3]. They reported results similar to our findings, namely that most US men had sex at least once per week with one partner and lower sexual activity among unemployed and unmarried men. The highest sexual activity was observed in 35-44-year-olds; men with low income or with part-time or no employment were more likely to be sexually inactive. In the UK, the Natsal-3 study interestingly revealed that the highest sexual activity was with young men, aged 25–34 [31]. Moreover, academic qualifications and professional occupations increased the likelihood of sexual intercourse. Further, in the UK English Longitudinal Study of Ageing, Jackson et al. specifically analysed the lifetime number of sexual partners; the Authors reported that young age, being separated/divorced or single/never married were independently associated with a high number of sexual partners [32]. In the Second Australian Study of Health and Relationships, Badcock et al. reported an average frequency of sex of 1.44 times per week and significantly more sexually active men were in relationships [33]. In Germany, 31-40-year-olds were the most sexually active group, and respondents living with a partner had more intercourse than those without a partner [8]. Therefore, despite slight differences between countries and regions, these studies showed a significant effect of sociodemographic parameters on men’s sexual activity. Because our observations appear broadly comparable with other reports, we can further confirm that sociodemographic variables, also in Poland, have an important effect on men’s sexuality, and importantly, we can speculate that the overall effect of these specific variables is quite independent from the countries or regions where the studies were performed.

Our study reaffirmed the negative effect of ED on men’s sexual relations. Notably, we detected a significant correlation between ED severity and effect on sex life, i.e., the more severe the ED, the lower sexual activity/the lower number of sexual partners. Our results agree with findings of Permpongkosol et al. who found that sexual dysfunction can compromise intimate relationships [34]. Therefore, although ED is often considered as a multidimensional disorder with many influencing factors (i.e., genetic, environmental, lifestyle, cultural), it seems that the effect of ED on men’s sex life may be largely independent of environmental or genetic influences. Conversely, in our analysis, PE did not affect the frequency of sexual activity or the number of sexual partners. Although PE may lower satisfaction of sexual intercourse and overall sexual relationship, our study did not agree with findings of other investigators who reported that PE led to less frequent intercourse. In 2007, in their community-based study of 1,587 men from the US, Rowland et al. demonstrated that respondents with PE reported significantly lower frequency of sexual intercourse [35]. Similar findings were presented by Peng et al. who demonstrated, again in a community analysis, that PE significantly correlated with the frequency of intercourse for young and middle-aged men in China (n = 923) [36]. In our study, with well-balanced demographic characteristics and a large representative sample size, PE did not decrease the intercourse frequency or the sexual partner number. Thus, we hypothesize that the effect of PE on men’s sexual activity might be subtle as opposed to the more obvious effect of ED.

We confirmed that frequency of sexual activity and number of sexual partners correlate with sex-specific and overall quality of life. In our analysis, the higher the frequency of sexual activity and the higher the number of sexual partners, the higher the sex-specific and the overall quality of life. Importantly, similar findings have been reported [37, 38], and the positive effect of sexual activity on quality of life seems to be independent of age, although the frequency of sexual activity decreases with age [6]. Cao et al. suggested a protective effect of sexual activity on enjoyment of life and well-being, and they found recently that sexual activity was associated with a lower risk of mortality from all cause and cancer [39]. In their analysis, participants with a high frequency of sexual activity were at a lower risk of all-cause death in a dose-response manner; further, the multivariable-adjusted hazard ratio for cancer mortality was 0.31 (CI 0.11–0.84) among participants who had sex at least 52 times/year compared with participants who had sex 0–1 time/year. Several mechanisms have been described to understand the positive associations between sexual activity and health [3]. Because sexual activity can be considered a form of physical activity, men who engage in regular sexual activity likely acquire the mental and physical health benefits from a physically active lifestyle [40]. During sexual activity or at the time of sexual intercourse, there is also a release of endorphins, i.e., neurotransmitters that block the perception of pain and increase feelings of happiness and wellbeing [41]. Further, these circulating endorphins correlate with greater activity of natural killer cells [42], cytotoxic lymphocytes that enable a rapid immune reaction by rapidly responding to viruses and other intracellular pathogens and even cancer [43]. Finally, people who engage in sexual intercourse are likely to share a closer relationship with their partners, and closeness to one’s partner is associated with well-being per se [44]. In conclusion, the association between sexual activity and physical and mental health is complex, and likely the corresponding mechanisms are not completely understood.

Multiple studies showed that people rarely seek medical help for sexual problems [45]. Analyses of barriers to seeking treatment have pointed to embarrassment, anxiety, social stigma, treatment cost, and a belief that sexual problems are a normal part of aging or a temporary dysfunction [46]. Often, people are simply unaware that there may be treatment for their ailment. In our analysis, one in ten men were looking for medical treatment for problems with their sex life. The most active treatment seekers were men with more than three partners in the last year and men who had intercourse less than once per month. We did not ask respondents why they were looking for medical help; thus, we can only hypothesize that the reasons were either mental (e.g., problems with the stability of relationships, especially for men with multiple partners; notably, the number of sexual partners in our study was significantly higher for men with borderline or significant distress that was screened with the HADS instrument) or physical (e.g., ED that could lower intercourse frequency, especially for men with already low frequency of sexual activity; notably, our analysis confirmed the profound effect of ED on men’s sexual activity). Even without these data, we need to underline that the knowledge about the lifetime of one’s sexual activity should serve as an important reference in counseling, indicating, and evaluating treatments for sexual dysfunction along the life trajectory of men. Appropriate educational initiatives, aimed at both patients and healthcare professionals, may help to increase awareness and understanding of men’s sexual health. Healthcare professionals should always consider identification and overcoming potential barriers that persons might have in discussing and seeking help for sexual problems.

An interesting observation from our study is the absence of any impact of analyzed comorbidities on intercourse frequency. However, lifestyle habits, i.e., smoking and alcohol intake, did limit sexual activity. Our results underline the significant influence of unhealthy behavior on healthy sexual functioning. Both smoking and alcohol intake have been proved risk factors for ED [45]. Some recent studies even showed that smoking, understood as both cigarette smoke and electronic nicotine delivery devices, has a negative dose-response effect on men’s erectile functioning [47–49]. In a study of a large cohort of middle-aged and older US adults, Chou et al. demonstrated that any lifetime and past-year substance-use disorder, alcohol and nicotine included, was significantly correlated with abstention from sexual intercourse [50]. Although use of alcohol, tobacco, and other drugs is predictive for early sexual initiation [51, 52], these substance in later life may significantly limit sexual activity [50] or even lead to severe mental distress [53]. Our findings further support this hypothesis.

The cross-sectional design was a main limitation of our study. With longitudinal analysis, we would be able to investigate trends in sexual activity among Polish men. As with all surveys that investigate a population, limitations also included the use of self-reports to measure sexual activity. Some respondents might not have been fully open or honest, especially with intimate information such as frequency of sexual activity and number of sexual partners. However, only a relatively small number of respondents did not provide clear answers by selecting ‘Hard to say/do not know’ when available as an option. In addition, we need to consider that sexual activity was not strictly defined in our survey as well as the use of pornography. Some participants may have interpreted sex and sex partners as vaginal intercourse (or sex partners as referring only to relational partners), whereas others may have considered sex to include oral sex or mutual masturbation [54, 55]. Men are more likely than women to report nonpenetrative sex as sex [54, 56]. Nevertheless, the discrepancies in interpretations of sex survey questions have been described and the significant information bias of population-based self-report data is inevitable for all surveys related to sexual activity [3, 54]. To circumvent these issues at least partially, we adapted questions of our survey related to the frequency of sexual activity and number of sexual partners from the American General Social Survey. We did not also investigate the sexual orientation of respondents. Finally, because this study was conducted in Poland, results may not be universally generalizable, especially for other ethnic groups. Nevertheless, cultural, linguistic, environmental, and, importantly, genetic homogeneity of Slavic people still exists at some point [57–61]; thus, our results might be considered as proxy for other Slavic populations and might have important implications for public health and societies of Central and Eastern Europe.

## Conclusions

This investigation was the first population-representative and nationwide study of frequency of sexual activity and number of sexual partners to be performed with men in Poland. Polish men are sexually active, with most men having sex at least once a week with one partner. All sociodemographic parameters had significant effects on men’s sexual activity. Men’s sexual relations correlated well with both sex-specific and overall quality of life and psychological distress. Erectile dysfunction and lifestyle habits significantly limited men’s sexual health, but other comorbidities, including premature ejaculation, did not affect sexual life.

## Supporting information

S1 Table Frequency of sexual activity and number of sexual partners in all age groups. (DOCX)

S2 Table Frequency of sexual activity and number of sexual partners across all 16 states/voivodships of Poland. (DOCX)

S3 Table Frequency of sexual activity and number of sexual partners as a function of education level. (DOCX)

S4 Table Frequency of sexual activity and number of sexual partners as a function of employment status. (DOCX)

S5 Table Frequency of sexual activity and number of sexual partners as a function of the IIEF-based severity categories for ED. (DOCX)

S6 Table Frequency of sexual activity and number of sexual partners as a function of the PEDT score. (DOCX)

S7 Table Frequency of sexual activity and number of sexual partners as a function of different scale’s cutoff points of PEDT. (DOCX)

S8 Table Frequency of sexual activity and number of sexual partners as a function of psychological distress based on HADS. (DOCX)
