# Supplementary material for: Sexual activity in a large representative cohort of Polish men: Frequency, number of partners, correlates, and quality of life
Source: PLoS One. 2024 Jan 19;19(1):e0296449. doi: 10.1371/journal.pone.0296449 (PMC10798542; doi:10.1371/journal.pone.0296449)
Supplement: S1 Table — (DOCX) [file pone.0296449.s001.docx]

S1 Table. Frequency of sexual activity and number of sexual partners in all age groups.

| **Parameter** | **Value** | **Age** | | | | | |  | **p** |
| --- | --- | --- | --- | --- | --- | --- | --- | --- | --- |
|  |  | **18-24**  **(N=309) - A** | **25-34**  **(N=586) - B** | **35-44**  **(N=703) - C** | **45-54**  **(N=549) - D** | **55-64**  **(N=578) - E** | **≥65**  **(N=276) - F** | **Overall**  **(N=3001)** |  |
| Frequency of sexual  activity in the past year | Not at all | 100 (32.36%) | 105 (17.92%) | 77 (10.95%) | 72 (13.11%) | 93 (16.09%) | 80 (28.99%) | 527 (17.56%) | p<0.001 |
|  | Less than once per month | 33 (10.68%) | 42 (7.17%) | 66 (9.39%) | 53 (9.65%) | 60 (10.38%) | 39 (14.13%) | 293 (9.76%) | B,E>A,F C>D,B,E,A,F D>E,A,F |
|  | 1-3 times per month | 64 (20.71%) | 132 (22.53%) | 156 (22.19%) | 142 (25.87%) | 160 (27.68%) | 81 (29.35%) | 735 (24.49%) |  |
|  | Weekly or more | 98 (31.72%) | 275 (46.93%) | 365 (51.92%) | 250 (45.54%) | 227 (39.27%) | 62 (22.46%) | 1277 (42.55%) |  |
|  | Hard to say | 14 (4.53%) | 32 (5.46%) | 39 (5.55%) | 32 (5.83%) | 38 (6.57%) | 14 (5.07%) | 169 (5.63%) |  |
| Number of sexual partners  in the past year | 0 | 100 (32.36%) | 103 (17.58%) | 76 (10.81%) | 72 (13.11%) | 90 (15.57%) | 80 (28.99%) | 521 (17.36%) | p<0.001 |
|  | 1 | 110 (35.60%) | 329 (56.14%) | 472 (67.14%) | 400 (72.86%) | 401 (69.38%) | 165 (59.78%) | 1877 (62.55%) | B,C>A,D,E>F |
|  | 2 | 35 (11.33%) | 47 (8.02%) | 52 (7.40%) | 31 (5.65%) | 34 (5.88%) | 10 (3.62%) | 209 (6.96%) |  |
|  | ≥3 | 59 (19.09%) | 95 (16.21%) | 84 (11.95%) | 37 (6.74%) | 38 (6.57%) | 16 (5.80%) | 329 (10.96%) |  |
|  | Hard to say | 5 (1.62%) | 12 (2.05%) | 19 (2.70%) | 9 (1.64%) | 15 (2.60%) | 5 (1.81%) | 65 (2.17%) |  |

p - Kruskal-Wallis test + post-hoc (Dunn test)
