# Supplementary material for: Sexual activity in a large representative cohort of Polish men: Frequency, number of partners, correlates, and quality of life
Source: PLoS One. 2024 Jan 19;19(1):e0296449. doi: 10.1371/journal.pone.0296449 (PMC10798542; doi:10.1371/journal.pone.0296449)
Supplement: S2 Table — (DOCX) [file pone.0296449.s002.docx]

S2 Table. Frequency of sexual activity and number of sexual partners across all 16 states/voivodships of Poland.

| **Parameter** | **Value** | **State/Voivodship** | | | | | | | | | | | | | | | | **p** |
| --- | --- | --- | --- | --- | --- | --- | --- | --- | --- | --- | --- | --- | --- | --- | --- | --- | --- | --- |
|  |  | **Dolnośląskie (N=211)** | **Kujawsko-pomorskie (N=173)** | **Lubelskie (N=185)** | **Lubuskie (N=97)** | **Łódzkie (N=182)** | **Małopolskie (N=216)** | **Mazowieckie (N=416)** | **Opolskie (N=73)** | **Podkarpackie (N=185)** | **Podlaskie (N=96)** | **Pomorskie (N=178)** | **Śląskie (N=377)** | **Świętokrzyskie (N=107)** | **Warmińsko-mazurskie (N=122)** | **Wielkopolskie (N=266)** | **Zachodniopomorskie (N=117)** |  |
| Frequency of sexual  activity in the past year | Not at all | 41 (19.43%) | 38 (21.97%) | 32 (17.30%) | 19 (19.59%) | 27 (14.84%) | 43 (19.91%) | 71 (17.07%) | 13 (17.81%) | 34 (18.38%) | 18 (18.75%) | 32 (17.98%) | 62 (16.45%) | 16 (14.95%) | 18 (14.75%) | 51 (19.17%) | 12 (10.26%) | p=0.49 |
|  | Less than once per month | 27 (12.80%) | 16 (9.25%) | 24 (12.97%) | 4 (4.12%) | 19 (10.44%) | 20 (9.26%) | 49 (11.78%) | 7 (9.59%) | 18 (9.73%) | 9 (9.38%) | 18 (10.11%) | 31 (8.22%) | 5 (4.67%) | 11 (9.02%) | 25 (9.40%) | 10 (8.55%) |  |
|  | 1-3 times per month | 49 (23.22%) | 42 (24.28%) | 41 (22.16%) | 30 (30.93%) | 51 (28.02%) | 46 (21.30%) | 95 (22.84%) | 21 (28.77%) | 40 (21.62%) | 24 (25.00%) | 44 (24.72%) | 94 (24.93%) | 36 (33.64%) | 31 (25.41%) | 67 (25.19%) | 24 (20.51%) |  |
|  | Weekly or more | 85 (40.28%) | 67 (38.73%) | 76 (41.08%) | 40 (41.24%) | 71 (39.01%) | 91 (42.13%) | 178 (42.79%) | 28 (38.36%) | 83 (44.86%) | 37 (38.54%) | 75 (42.13%) | 175 (46.42%) | 46 (42.99%) | 55 (45.08%) | 109 (40.98%) | 61 (52.14%) |  |
|  | Hard to say | 9 (4.27%) | 10 (5.78%) | 12 (6.49%) | 4 (4.12%) | 14 (7.69%) | 16 (7.41%) | 23 (5.53%) | 4 (5.48%) | 10 (5.41%) | 8 (8.33%) | 9 (5.06%) | 15 (3.98%) | 4 (3.74%) | 7 (5.74%) | 14 (5.26%) | 10 (8.55%) |  |
| Number of sexual partners in the past year | 0 | 40 (18.96%) | 37 (21.39%) | 37 (20.00%) | 20 (20.62%) | 25 (13.74%) | 44 (20.37%) | 75 (18.03%) | 16 (21.92%) | 33 (17.84%) | 18 (18.75%) | 30 (16.85%) | 57 (15.12%) | 16 (14.95%) | 19 (15.57%) | 43 (16.17%) | 11 (9.40%) | p=0.701 |
|  | 1 | 124 (58.77%) | 103 (59.54%) | 115 (62.16%) | 55 (56.70%) | 125 (68.68%) | 130 (60.19%) | 257 (61.78%) | 37 (50.68%) | 107 (57.84%) | 56 (58.33%) | 101 (56.74%) | 253 (67.11%) | 70 (65.42%) | 89 (72.95%) | 174 (65.41%) | 81 (69.23%) |  |
|  | 2 | 18 (8.53%) | 9 (5.20%) | 8 (4.32%) | 9 (9.28%) | 8 (4.40%) | 10 (4.63%) | 35 (8.41%) | 6 (8.22%) | 12 (6.49%) | 9 (9.38%) | 19 (10.67%) | 30 (7.96%) | 9 (8.41%) | 5 (4.10%) | 13 (4.89%) | 9 (7.69%) |  |
|  | ≥3 | 24 (11.37%) | 16 (9.25%) | 23 (12.43%) | 11 (11.34%) | 19 (10.44%) | 26 (12.04%) | 41 (9.86%) | 12 (16.44%) | 31 (16.76%) | 11 (11.46%) | 27 (15.17%) | 30 (7.96%) | 8 (7.48%) | 9 (7.38%) | 30 (11.28%) | 11 (9.40%) |  |
|  | Hard to say | 5 (2.37%) | 8 (4.62%) | 2 (1.08%) | 2 (2.06%) | 5 (2.75%) | 6 (2.78%) | 8 (1.92%) | 2 (2.74%) | 2 (1.08%) | 2 (2.08%) | 1 (0.56%) | 7 (1.86%) | 4 (3.74%) | 0 (0.00%) | 6 (2.26%) | 5 (4.27%) |  |

p - Kruskal-Wallis test
