# Supplementary material for: Sexual activity in a large representative cohort of Polish men: Frequency, number of partners, correlates, and quality of life
Source: PLoS One. 2024 Jan 19;19(1):e0296449. doi: 10.1371/journal.pone.0296449 (PMC10798542; doi:10.1371/journal.pone.0296449)
Supplement: S3 Table — (DOCX) [file pone.0296449.s003.docx]

S3 Table. Frequency of sexual activity and number of sexual partners as a function of education level.

| **Parameter** | **Value** | **Education** | | | | **p** |
| --- | --- | --- | --- | --- | --- | --- |
|  |  | **Elementary (N=87) - A** | **Vocational (N=393) - B** | **Secondary (N=1343) - C** | **Higher (N=1178) - D** |  |
| Frequency of sexual  activity in the past year | Not at all | 30 (34.48%) | 72 (18.32%) | 243 (18.09%) | 182 (15.45%) | p<0.001 |
|  | Less than once per month | 14 (16.09%) | 44 (11.20%) | 131 (9.75%) | 104 (8.83%) | C,B>A D>B,A |
|  | 1-3 times per month | 17 (19.54%) | 98 (24.94%) | 301 (22.41%) | 319 (27.08%) |  |
|  | Weekly or more | 20 (22.99%) | 153 (38.93%) | 576 (42.89%) | 528 (44.82%) |  |
|  | Hard to say | 6 (6.90%) | 26 (6.62%) | 92 (6.85%) | 45 (3.82%) |  |
| Number of sexual partners in the past year | 0 | 29 (33.33%) | 82 (20.87%) | 237 (17.65%) | 173 (14.69%) | p=0.083 |
|  | 1 | 38 (43.68%) | 223 (56.74%) | 824 (61.36%) | 792 (67.23%) |  |
|  | 2 | 6 (6.90%) | 26 (6.62%) | 95 (7.07%) | 82 (6.96%) |  |
|  | ≥3 | 10 (11.49%) | 52 (13.23%) | 153 (11.39%) | 114 (9.68%) |  |
|  | Hard to say | 4 (4.60%) | 10 (2.54%) | 34 (2.53%) | 17 (1.44%) |  |

p - Kruskal-Wallis test + post-hoc (Dunn test)
