# Supplementary material for: Sexual activity in a large representative cohort of Polish men: Frequency, number of partners, correlates, and quality of life
Source: PLoS One. 2024 Jan 19;19(1):e0296449. doi: 10.1371/journal.pone.0296449 (PMC10798542; doi:10.1371/journal.pone.0296449)
Supplement: S4 Table — (DOCX) [file pone.0296449.s004.docx]

S4 Table. Frequency of sexual activity and number of sexual partners as a function of employment status.

| **Parameter** | **Value** | **Employment** | | | | **p** |
| --- | --- | --- | --- | --- | --- | --- |
|  |  | **Employed**  **(N=2199) - A** | **Unemployed**  **(N=292) - B** | **Pensioner**  **(N=444) - C** | **Other**  **(N=66) - D** |  |
| Frequency of sexual  activity in the past year | Not at all | 262 (11.91%) | 129 (44.18%) | 125 (28.15%) | 11 (16.67%) | p<0.001 |
|  | Less than once per month | 204 (9.28%) | 34 (11.64%) | 48 (10.81%) | 7 (10.61%) | A,D>C>B |
|  | 1-3 times per month | 532 (24.19%) | 56 (19.18%) | 132 (29.73%) | 15 (22.73%) |  |
|  | Weekly or more | 1083 (49.25%) | 59 (20.21%) | 108 (24.32%) | 27 (40.91%) |  |
|  | Hard to say | 118 (5.37%) | 14 (4.79%) | 31 (6.98%) | 6 (9.09%) |  |
| Number of sexual partners in the past year | 0 | 255 (11.60%) | 126 (43.15%) | 128 (28.83%) | 12 (18.18%) | p<0.001 |
|  | 1 | 1473 (66.98%) | 105 (35.96%) | 271 (61.04%) | 28 (42.42%) | D,A>B,C |
|  | 2 | 162 (7.37%) | 19 (6.51%) | 24 (5.41%) | 4 (6.06%) |  |
|  | ≥3 | 261 (11.87%) | 36 (12.33%) | 13 (2.93%) | 19 (28.79%) |  |
|  | Hard to say | 48 (2.18%) | 6 (2.05%) | 8 (1.80%) | 3 (4.55%) |  |

p - Kruskal-Wallis test + post-hoc (Dunn test)
