# Supplementary material for: Sexual activity in a large representative cohort of Polish men: Frequency, number of partners, correlates, and quality of life
Source: PLoS One. 2024 Jan 19;19(1):e0296449. doi: 10.1371/journal.pone.0296449 (PMC10798542; doi:10.1371/journal.pone.0296449)
Supplement: S5 Table — (DOCX) [file pone.0296449.s005.docx]

S5 Table. Frequency of sexual activity and number of sexual partners as a function of the IIEF-based severity categories for ED.

| **Parameter** | **Value** | **IIEF** | | | | | **p** |
| --- | --- | --- | --- | --- | --- | --- | --- |
|  |  | **Severe ED**  **(N=96) - A** | **Moderate ED**  **(N=200) - B** | **Mild to moderate ED**  **(N=606) - C** | **Mild ED**  **(N=932) - D** | **Without ED**  **(N=1167) - E** |  |
| Frequency of sexual  activity in the past year | Not at all | 71 (73.96%) | 87 (43.50%) | 87 (14.36%) | 138 (14.81%) | 144 (12.34%) | p<0.001 |
|  | Less than once per month | 12 (12.50%) | 38 (19.00%) | 88 (14.52%) | 77 (8.26%) | 78 (6.68%) | E>D>C>B>A |
|  | 1-3 times per month | 8 (8.33%) | 44 (22.00%) | 200 (33.00%) | 254 (27.25%) | 229 (19.62%) |  |
|  | Weekly or more | 3 (3.12%) | 25 (12.50%) | 199 (32.84%) | 410 (43.99%) | 640 (54.84%) |  |
|  | Hard to say | 2 (2.08%) | 6 (3.00%) | 32 (5.28%) | 53 (5.69%) | 76 (6.51%) |  |
| Number of sexual partners in the past year | 0 | 68 (70.83%) | 83 (41.50%) | 91 (15.02%) | 136 (14.59%) | 143 (12.25%) | p<0.001 |
|  | 1 | 22 (22.92%) | 85 (42.50%) | 352 (58.09%) | 607 (65.13%) | 811 (69.49%) | C>D,E>B>A |
|  | 2 | 1 (1.04%) | 16 (8.00%) | 50 (8.25%) | 67 (7.19%) | 75 (6.43%) |  |
|  | ≥3 | 3 (3.12%) | 15 (7.50%) | 102 (16.83%) | 107 (11.48%) | 102 (8.74%) |  |
|  | Hard to say | 2 (2.08%) | 1 (0.50%) | 11 (1.82%) | 15 (1.61%) | 36 (3.08%) |  |

p - Kruskal-Wallis test + post-hoc (Dunn test)
