# Supplementary material for: Sexual activity in a large representative cohort of Polish men: Frequency, number of partners, correlates, and quality of life
Source: PLoS One. 2024 Jan 19;19(1):e0296449. doi: 10.1371/journal.pone.0296449 (PMC10798542; doi:10.1371/journal.pone.0296449)
Supplement: S6 Table — (DOCX) [file pone.0296449.s006.docx]

S6 Table. Frequency of sexual activity and number of sexual partners as a function of the PEDT score.

| **Parameter** | **Value** | **PEDT** | | **p** |
| --- | --- | --- | --- | --- |
|  |  | **PEDT score: 10 and less**  **(N=2423)** | **PEDT score: 11 and more**  **(N=578)** |  |
| Frequency of sexual  activity in the past year | Not at all | 433 (17.87%) | 94 (16.26%) | p=0.184 |
|  | Less than once per month | 234 (9.66%) | 59 (10.21%) |  |
|  | 1-3 times per month | 562 (23.19%) | 173 (29.93%) |  |
|  | Weekly or more | 1058 (43.66%) | 219 (37.89%) |  |
|  | Hard to say | 136 (5.61%) | 33 (5.71%) |  |
| Number of sexual partners in the past year | 0 | 430 (17.75%) | 91 (15.74%) | p=0.761 |
|  | 1 | 1497 (61.78%) | 380 (65.74%) |  |
|  | 2 | 177 (7.30%) | 32 (5.54%) |  |
|  | ≥3 | 264 (10.90%) | 65 (11.25%) |  |
|  | Hard to say | 55 (2.27%) | 10 (1.73%) |  |

p - Mann-Whitney test
