# Supplementary material for: Sexual activity in a large representative cohort of Polish men: Frequency, number of partners, correlates, and quality of life
Source: PLoS One. 2024 Jan 19;19(1):e0296449. doi: 10.1371/journal.pone.0296449 (PMC10798542; doi:10.1371/journal.pone.0296449)
Supplement: S7 Table — (DOCX) [file pone.0296449.s007.docx]

S7 Table. Frequency of sexual activity and number of sexual partners as a function of different scale’s cutoff points of PEDT.

| **Parameter** | **Value** | **PEDT** | | | **p** |
| --- | --- | --- | --- | --- | --- |
|  |  | **Without PE**  **(N=1859)** | **Probable presence of PE**  **(N=564)** | **Presence of PE**  **(N=578)** |  |
| Frequency of sexual  activity in the past year | Not at all | 341 (18.34%) | 92 (16.31%) | 94 (16.26%) | p=0.069 |
|  | Less than once per month | 167 (8.98%) | 67 (11.88%) | 59 (10.21%) |  |
|  | 1-3 times per month | 409 (22.00%) | 153 (27.13%) | 173 (29.93%) |  |
|  | Weekly or more | 844 (45.40%) | 214 (37.94%) | 219 (37.89%) |  |
|  | Hard to say | 98 (5.27%) | 38 (6.74%) | 33 (5.71%) |  |
| Number of sexual partners in the past year | 0 | 337 (18.13%) | 93 (16.49%) | 91 (15.74%) | p=0.061 |
|  | 1 | 1164 (62.61%) | 333 (59.04%) | 380 (65.74%) |  |
|  | 2 | 125 (6.72%) | 52 (9.22%) | 32 (5.54%) |  |
|  | ≥3 | 191 (10.27%) | 73 (12.94%) | 65 (11.25%) |  |
|  | Hard to say | 42 (2.26%) | 13 (2.30%) | 10 (1.73%) |  |

p - Kruskal-Wallis test
