# Supplementary material for: Sexual activity in a large representative cohort of Polish men: Frequency, number of partners, correlates, and quality of life
Source: PLoS One. 2024 Jan 19;19(1):e0296449. doi: 10.1371/journal.pone.0296449 (PMC10798542; doi:10.1371/journal.pone.0296449)
Supplement: S8 Table — (DOCX) [file pone.0296449.s008.docx]

S8 Table. Frequency of sexual activity and number of sexual partners as a function of psychological distress based on HADS.

| **Parameter** | **Value** | **HADS** | | | **p** |
| --- | --- | --- | --- | --- | --- |
|  |  | **No distress**  **(N=1530) - A** | **Borderline distress**  **(N=676) - B** | **Significant distress**  **(N=795) - C** |  |
| Frequency of sexual  activity in the past year | Not at all | 230 (15.03%) | 129 (19.08%) | 168 (21.13%) | p<0.001 |
|  | Less than once per month | 130 (8.50%) | 76 (11.24%) | 87 (10.94%) | A>B,C |
|  | 1-3 times per month | 384 (25.10%) | 163 (24.11%) | 188 (23.65%) |  |
|  | Weekly or more | 706 (46.14%) | 263 (38.91%) | 308 (38.74%) |  |
|  | Hard to say | 80 (5.23%) | 45 (6.66%) | 44 (5.53%) |  |
| Number of sexual partners in the past year | 0 | 241 (15.75%) | 126 (18.64%) | 154 (19.37%) | p<0.001 |
|  | 1 | 1079 (70.52%) | 372 (55.03%) | 426 (53.58%) | C,B>A |
|  | 2 | 94 (6.14%) | 60 (8.88%) | 55 (6.92%) |  |
|  | ≥3 | 87 (5.69%) | 102 (15.09%) | 140 (17.61%) |  |
|  | Hard to say | 29 (1.90%) | 16 (2.37%) | 20 (2.52%) |  |

p - Kruskal-Wallis test + post-hoc (Dunn test)
